# Self‐report screening instruments differentiate bipolar disorder and borderline personality disorder

**DOI:** 10.1002/brb3.2201

**Published:** 2021-05-30

**Authors:** Brian A. Palmer, Mehak Pahwa, Jennifer R. Geske, Simon Kung, Malik Nassan, Kathryn M. Schak, Renato D. Alarcon, Mark A. Frye, Balwinder Singh

**Affiliations:** ^1^ Department of Psychiatry and Psychology Mayo Clinic Rochester MN USA; ^2^ Mental Health and Addiction Clinical Service Line Allina Health Minneapolis MN USA; ^3^ Department of Quantitative Health Services Mayo Clinic Rochester MN USA; ^4^ School of Medicine Universidad Peruana Cayetano Heredia Lima Peru

**Keywords:** bipolar disorder, borderline personality disorder, MDQ, MSI

## Abstract

**Background:**

Bipolar disorder (BD) and borderline personality disorder (BPD) share overlapping phenomenology and are frequently misdiagnosed. This study investigated the diagnostic accuracy of the Mood Disorder Questionnaire (MDQ) and McLean Screening Instrument for Borderline Personality Disorder (MSI) in a clinical inpatient setting and whether individual screening items could differentiate BD from BPD.

**Methods:**

757 sequential inpatients admitted to a Mood Disorder Unit completed both the MDQ and MSI. Screen positive for the MDQ was defined as ≥7/13 symptoms endorsed with concurrence and at least moderate impact. Screen positive for the MSI was defined as a score of ≥7. The clinical discharge summary diagnosis completed by a board‐certified psychiatrist was used as the reference standard to identify concordance rates of a positive screen with clinical diagnosis. Individual items predicting one disorder and simultaneously predicting absence of other disorder by odds ratio (OR>and <1) were identified.

**Results:**

Both screening instruments were more specific than sensitive (MDQ 83.7%/ 67.8%, MSI 73.2% / 63.3%). MDQ individual items (elevated mood, grandiosity, increased energy, pressured speech, decreased need for sleep, hyperactivity) were significant predictors of BD diagnosis and non‐predictors of BPD diagnosis. Whereas MSI subitem, self‐harm behaviors/suicidal attempts predicted BPD in the absence of BD; distrust and irritability were additional predictors of BPD.

**Conclusion:**

While this study is limited by the lack of structured diagnostic interview, these data provide differential symptoms to discriminate BD and BPD. Further work with larger datasets and more rigorous bioinformatics machine learning methodology is encouraged to continue to identify distinguishing features of these two disorders to guide diagnostic precision and subsequent treatment recommendations.

## Graphical abstract

1. Clinical differentiation between bipolar disorder and borderline personality disorder can be challenging as both disorders share certain features like impulsivity, mood lability, and affective instability.
2. The results from this study offer practical and useful clinical guidance for focusing on specific sub‐items from the Mood Disorder Questionnaire (MDQ) and McLean Screening Instrument for borderline personality disorder (MSI) that can help distinguish bipolar disorder and borderline personality disorder.

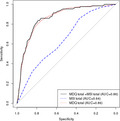

## 1 INTRODUCTION

Clinical differentiation between bipolar disorder (BD) and borderline personality disorder (BPD) can be challenging (Bolton & Gunderson, 1996; Paris, 2004; Paris & Zweig‐Frank, 2001). Both disorders share certain symptom domains of impulsivity, mood lability, and affective instability (AI). Given this phenomenologic overlap, there has been longstanding, and as yet, unresolved debate whether these disorders should be viewed as a spectrum or continuum or be viewed as having diagnostic separation (Kernberg & Yeomans, 2013; Siever & Davis, 1991; Zimmerman & Morgan, 2013). A recent study showed a genetic overlap between the two disorders, but, these data need further exploration (Witt et al., 2017).

In a comprehensive review of 24 BD studies (including inpatient and outpatients), the prevalence of BPD was 16% (Zimmerman & Morgan, 2013). While some evidence suggests a higher prevalence of BPD in BD‐II than in BD‐I, the only two studies which compared the disorders directly did not find a significant difference (Vieta et al., 2000; Zimmerman & Mattia, 1999). The same comprehensive review reported the prevalence of BD was 14.1% in patients with BPD (Zimmerman & Morgan, 2013). Among studies that reports rates of both BD‐I and BD‐II, 9.3% had BD‐I diagnosis, and 10.1% had BD‐II. Taken together, this suggests that overall there is an approximately 15% prevalence of BD‐BPD comorbidity (Zimmerman & Morgan, 2013). When the comorbidity is present, bipolar course of illness measures are increased (i.e., episode frequency, affective instability, impulsivity, and self‐mutilation/suicidality) (Riemann et al., 2017). Despite the high rates of co‐occurrence, a key clinical finding from longitudinal studies suggests that BD comorbidity has no/minimal significant impact on BPD course (global functioning, clinical course, and response to treatment) (Frias et al., 2016). However, BPD has a modest impact on BD course (Gunderson et al., 2006, 2011), suggesting that these comorbid disorders, each merit their own appropriate treatment, even when they co‐occur. Thus, accurately diagnosing BPD and BD in clinical setting is essential, given the impact of diagnosis on management; best practices would focus on mood stabilizers (primarily) and psychotherapy (secondary) for BD and psychotherapy (primary) for BPD. The implications from a treatment perspective are more magnified in an inpatient setting (Ghaemi et al., 2014).

The clinical assessment can be informed by the phenomenological literature, addressing key aspects of the relevant clinical presentation. The first construct, AI is characterized by mood changes, temporal instability, high intensity, and delayed recovery from dysphoria (Koenigsberg, 2010). AI is shared by both BPD and BP, but with different patterns (Henry et al., 2001). The shift from anger to euthymia is associated with BPD, whereas the shift from euthymia to depression or mood elation is associated with BD (Reich et al., 2012). Impulsivity is also frequent in both disorders. When comparing both disorders, BPD and BD‐II, using Barratt Impulsiveness Scale (BIS), higher scores for impulsiveness were reported in BPD versus BD‐II (Henry et al., 2001; Wilson et al., 2007). However, the highest BIS scores were found in a group that had both diagnoses BD‐II +BPD, suggesting a compound effect of comorbidity. Subjective mood experiences are a third area to consider phenomenologically. Depressive states in BPD are often characterized by emptiness, shame, and painful incoherence compared with the decreased self‐esteem and self‐criticism in BD (Bayes et al., 2014; Meares et al., 2011). Mood episodes tend to be more spontaneous in BD, whereas symptoms of BPD are usually reactive and triggered by an interpersonal event such as abandonment, rejection, or frustration (Kernberg & Yeomans, 2013; Paris et al., 2007; Renaud et al., 2012). Psychotic symptoms can occur in both disorders, however with different patterns. In BD, psychotic episodes are longer in duration but rarely extend beyond several months. BPD patients experience transient dissociative and paranoid symptoms than can be reactive to stressors (Adams & Sanders, 2011; Barnow et al., 2010; Glaser et al., 2010; Goodwin., 2007; Skodol et al., 2002).

Given the emergence of screening questionnaires playing an increasingly larger role in clinical practice, and the importance of accurate diagnosis for management of these common and severe illnesses, utilizing validated self‐report standardized screening instruments may help improve diagnosing these disorders accurately. The objective of this study was to identify clinical features that can help differentiate BD versus BPD in an acute inpatient setting.

## 2 METHODS

This study was approved by the institutional review board at Mayo Clinic.

### 2.1 Participants

A consecutive sample of adult (≥ 18 years) inpatients admitted to a specialized Mood Disorders Unit at Mayo Clinic, Rochester, Minnesota were included over an 18 months period. From an initial sample of 1,002 discharges with valid research authorizations on file, 88 patients had multiple admissions (earliest admission with complete data was used), and 203 patients had incomplete data (eliminated from analysis), including 46 of those with multiple admissions. This resulted in 757 sequentially admitted patients who completed both the Mood Disorder Questionnaire (MDQ) and the McLean Screening Instrument for Borderline Personality Disorder (MSI). BD and BPD diagnoses were based on the Diagnostic and Statistical Manual of Mental Disorders (DSM‐IV TR) diagnostic criteria by structured interview. Discharge diagnoses by board‐certified psychiatrists at Mayo Clinic were recorded for each participant and were used as a reference standard to assess the diagnostic accuracy.

### 2.2 Screening instruments

The MDQ (Hirschfeld et al., 2000) is a self‐report screening instrument for BD and consists of 5 sections. Section 1 contains 13 items; each can be answered with yes or no. A total score of 7 “yes” with concurrence and at least moderate disability indicates a positive screen. The original MDQ validation study in outpatient psychiatric clinics reported a sensitivity of 73% and specificity of 90% (Hirschfeld et al., 2000). Subsequent studies in a U.S.‐population‐based sample reported 28% sensitivity and 97% specificity (Hirschfeld et al., 2003), whereas the sensitivity was 92% and specificity 64% on an inpatient Mood Disorders Unit (Kung et al., 2015)​. A meta‐analysis reported a sensitivity of 66% and specificity of 79% (Carvalho et al., 2015) in mental health settings and 43% sensitivity and 95% specificity in the Primary care or general population setting.

The MSI (Zanarini et al., 2003) is a self‐report screening instrument for BPD and consists of 10 items that can be answered with either “yes” or “no.” A total score of 7 or above is correlated with a positive screen. The validation of MSI in an outpatient setting indicated sensitivity of 81% and specificity of 85% (Zanarini et al., 2003). A recent meta‐analysis of 11 studies reported the overall sensitivity of the MSI as 80% and specificity of 66% at a cutoff point of 7 (Zimmerman, 2021). The sensitivity and specificity of MSI scale varied among some studies based on a higher cutoff point of >7 (Chanen et al., 2008; Kroger et al., 2011).

### 2.3 Statistical analyses

Using the discharge diagnosis of a board‐certified psychiatrist as the reference standard, sensitivity, specificity, positive, and negative predictive values for both screening instruments were calculated. Correlation analyses were completed with Pearson's correlation coefficient for continuous variables and *t*‐test for categorical variables. Individual screening items were entered into logistic regression models to test each item as individual predictors of each disorder [i.e., odds ratio (OR) >1] and simultaneously predicting absence of other disorder (OR <1) were identified. Receiver operating characteristic (ROC) curves were utilized to assess different prediction models (MDQ, MSI, and MDQ+MSI) for BD and BPD.

## 3 RESULTS

Among 757 sequentially admitted patients who completed both the MDQ and the MSI, the mean age was 40.8 ± 13.2 *SD* years and 67.4% were female. A total of 190 patients had positive screen on the MDQ and 225 had a positive screen on the MSI. Upon discharge, 130 patients had a clinical diagnosis of BD, 60 had BPD, 532 had unipolar major depression, and 95 did not receive any of the aforementioned diagnoses. Eighteen patients were diagnosed with comorbid BD‐BPD. Demographics, psychiatric comorbidities and prior number of hospital admissions are summarized in the supplement (Table S1). The prevalence of BPD in patients with BD was 13.9% and 7.7% in patients with unipolar major depression. The prevalence of psychotic disorders was 3%, and the rate of comorbid substance use disorder was 15%. Sensitivity and specificity were calculated for MDQ and MSI at the optimal cutoff in this sample, that is ≥7 (Table S2), which is consistent with the literature (Hirschfeld et al., 2003; Zimmerman, 2021). The sensitivity and specificity of the MDQ positive screen for a clinical diagnosis of BP at discharge were 68% and 84%, respectively. For the MSI positive screen, the sensitivity and specificity for a clinical diagnosis of BPD were 63% and 73%, respectively (Table 1).

**TABLE 1:** Sensitivity, Specificity, and the Concordance and Discordance between the screening instruments and the clinical BD and BPD diagnosis (*N* = 757)

	Bipolar Disorder			Borderline Personality Disorder	
	Present	Absent		Present	Absent
MDQ positive	88	102	MSI positive	38	187
MDQ negative	42	525	MSI negative	22	510
Sensitivity (95% C.I.)	67.69% (58.93% – 75.63%)		63.33% (49.90% to 75.41%)		
Specificity (95% C.I.)	83.73% (80.61% – 86.54%)		73.17% (69.72% to 76.43%)		
PPV (95% C.I.)	46.32% (41.07% – 51.65%)		16.89% (13.92% to 20.34%)		
NPV (95% C.I.)	92.59% (90.67% – 94.14%)		95.86% (94.31% to 97.01%)		

Abbreviations: BD, Bipolar Disorder; BPD, borderline personality disorder; MDQ, Mood Disorder Questionnaire; MSI, McLean Screening Instrument for Borderline Personality Disorder; NPV, negative predictive value; PPV, positive predictive value.

In an attempt to identify specific symptoms that might help differentiate BD from BPD, MDQ and MSI subitems were analyzed individually for their prediction of BD or BPD (Table 2 and Table 3). Six MDQ subitems (elevated mood, grandiosity, decreased need for sleep, pressured speech, increased energy, and hyperactivity) were statistically significant in predicting the presence of BD (OR>1) and absence of clinical diagnosis of BPD (OR<1) [Table 2]. MSI subitem self‐harm behaviors/suicidal attempts was statistically significant in predicting the presence of BPD (OR>1) and the absence of BD diagnosis (OR<1) [Table 3]. Individual level data for MDQ and MSI scales are reported in eTable 3.

**TABLE 2:** MDQ subitems predicting diagnosis of bipolar disorder and absence of borderline personality disorder (*n* = 60 BPD, *n* = 130 BD, *n* = 18 comorbid BPD+BD; n total=172)

MDQ Subitem	Predicting BPD *n* = 60/172		Predicting Bipolar Disorder *n* = 130/172	
	OR (95% CI)	p‐value	OR (95% CI)	p‐value
**1. Elevated/ Expansive Mood**	0.31 (0.16, 0.60)	**0.0005**	**7.10 (3.26, 15.50)**	**<0.0001**
2. Irritability	2.05 (0.98, 4.32)	0.0578	0.70 (0.32, 1.57)	0.3894
**3. Increased Self Esteem**	0.30 (0.16, 0.58)	**0.0003**	**7.35 (3.38, 15.98)**	**<0.0001**
**4. Decreased Sleep**	0.33 (0.17, 0.64)	**0.0011**	**5.60 (2.65, 11.83)**	**<0.0001**
**5. Talkativeness**	0.26 (0.13, 0.53)	**0.0002**	**8.59 (3.91, 18.89)**	**<0.0001**
6. Racing Thoughts	0.43 (0.16, 1.12)	0.0842	3.24 (1.13, 8.64)	**0.0185**
7. Distractibility	1.13 (0.46, 2.80)	0.7914	1.58 (0.63, 4.00)	0.3307
**8. Increased Energy**	0.23 (0.11, 0.46)	**<0.0001**	**8.36 (3.84, 18.21)**	**<0.0001**
**9. Hyperactivity**	0.27 (0.14, 0.53)	**0.0002**	**7.49 (3.77, 16.13)**	**<0.0001**
10. Increase in social activity	0.62 (0.33, 1.19)	0.1497	4.25 (1.83, 9.88)	**0.0008**
11. Increased Sexual Drive	0.67 (0.36, 1.26)	0.2148	2.05 (1.01, 4.19)	**0.0481**
12. Excessive involvement in unusual/foolish/risky activities	0.59 (0.31, 1.14)	0.1158	3.35 (1.63, 6.89)	**0.0010**
13. Increased Spending	0.97 (0.52, 1.81)	0.9111	1.51 (0.75, 3.04)	0.2510

Note

**Bold**: *p* <.05.

Abbreviations: BPD, borderline personality disorder; CI, confidence interval;MDQ, Mood Disorder Questionnaire.

**TABLE 3:** MSI subitems predicting diagnosis of borderline personality disorder in absence of bipolar disorder. (*n* = 60 BPD, *n* = 130 BD, *n* = 18 comorbid BPD+BD; n total=172)

MSI Subitem	Predicting BPD *n* = 60/172		Predicting Bipolar Disorder *n* = 130/172	
	OR (95% CI)	p‐value	OR (95% CI)	p‐value
**1.** Unstable Relationships	1.22 (0.63, 2.34)	0.5584	1.34 (0.66, 2.73)	0.4242
**2. Self‐Harm/ Suicide attempt**	**4.38 (2.10, 9.12)**	**<0.0001**	0.25 (0.11, 0.59)	**0.0015**
**3.** Impulsivity	1.58 (0.77, 3.23)	0.2138	0.96 (0.44, 2.07)	0.9131
**4.** Unstable Mood	1.25 (0.53, 2.94)	0.6162	1.21 (0.49, 2.98)	0.6782
5. Anger outburst	1.83 (0.92, 3.64)	0.0844	0.68 (0.32, 1.45)	0.3125
**6. Distrustful**	**2.06 (1.01, 4.20)**	**0.0461**	0.76 (0.36, 1.64)	0.4884
**7.** Dissociative Symptoms	1.02 (0.53, 1.96)	0.9561	1.99 (0.92, 4.31)	0.0805
**8.** Chronic Feeling of Emptiness	1.71 (0.74, 3.93)	0.2110	1.08 (0.46, 2.55)	0.8573
**9.** Identity Disturbance	1.29 (0.68, 2.43)	0.4385	0.91 (0.45, 1.83)	0.7947
**10**. Frantic Efforts to Avoid Abandonment	1.48 (0.78, 2.78)	0.2281	1.05 (0.52, 2.12)	0.8858

Note

**Bold**: *p* <.05.

Abbreviations: BPD =borderline personality disorder; MSI=McLean Screening Instrument for Borderline Personality Disorder.

**FIGURE 1:**
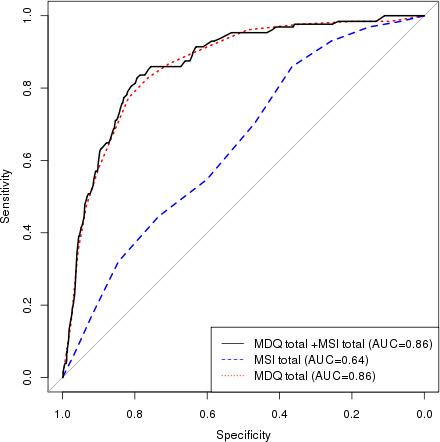
Model performance ROC curves of MDQ, MSI, and MDQ+MSI predicting Bipolar disorder

**FIGURE 2:**
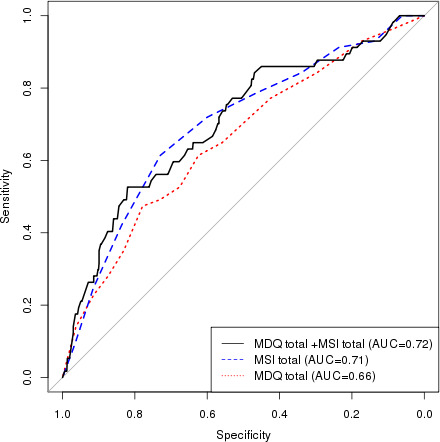
Model performance ROC curves of MDQ, MSI, and MDQ+MSI predicting Borderline Personality Disorder

Utilizing ROC curves, we tested different prediction models (MDQ, MSI, and MDQ+MSI) for BD and BPD. For BD, adding MSI to MDQ mildly raised the accuracy of the model, area under the curve (AUC) from 0.858 (MDQ) to 0.862 (MSI+MDQ) [Figure 1]. For BPD, adding MDQ to MSI minimally raised the accuracy of the model from 0.712 (MSI) to 0.717 (MSI+MDQ) [Figure 2]. Utilizing the significant subitems (MDQ: 1, 3, 4, 5, 8, and 9, MSI: 2) that differentiated between BD and BPD in a multivariable model, the ROC‐AUC for predicting BD in the total sample was 0.88 and for predicting BPD was 0.67 (Figure S1).

## 4 DISCUSSION

This study utilized validated self‐report standardized screening instruments in comparison to clinical diagnosis to identify clinical features that differentiate BD versus BPD in a large cohort of patients assessed in an acute setting. The result is a clinically useful roadmap to use screening questions to help differentiate BD and BPD. MDQ subitem analysis showed that elevated mood, grandiosity, increased energy, pressured speech, decreased need for sleep, and hyperactivity were significant in predicting BD and absence of BPD simultaneously. In addition, racing thoughts, increased social activities, sexual, and risky behaviors were predictors of BD. MSI subitem analysis showed that self‐harm behaviors (e.g., punching yourself, cutting yourself, burning yourself) and/or previous suicide attempts predicted BPD and absence of BD. Moreover, feeling distrustful of other people was a predictor of BPD. Irritability on the MDQ was more associated with BPD than BD. The remaining items of both the scales were not significant in predicting either BD or BPD. In simple terms, a *history of periods of sleep‐deprived energy enhancement with an elevated mood/grandiosity, increased rate of speech, and goal‐directed hyperactivity* can help define a ***BD*** diagnosis and exclude BPD diagnosis, while *self‐injury* (excludes BD diagnosis), *mistrust in relationships, and anger/irritability* can help identify ***BPD*** diagnosis.

A comprehensive assessment remains the goal standard for the diagnosis of BD and BPD in the absence of biomarkers. However, identifying items from validated self‐report standardized screening instruments may help clinicians narrow down clinical phenotypes and can help screen patients for inclusion in research studies. An accurate diagnosis of BD, BPD, or co‐occurrence can help guide treatment. Lithium treatment, for example, shows no clear utility for BPD (Bellino et al., 2011; Gunderson, 2009) while its efficacy for BD is unequivocal. When the disorders are comorbid, it may be wise to consider the beneficial effects with atypical antipsychotics and mood stabilizers (known to be effective in treating BD) for treating affective dysregulation and impulsive‐behavioral dyscontrol in BPD (Vita et al., 2011). There are no approved pharmacological treatment that showed efficacy in treating the core symptoms of BPD (Stoffers et al., 2010; Pahwa et al., 2020), and medications for this condition are at best adjunctive, with psychosocial and psychotherapeutic approaches offering the most effective treatment (Paris & Black, 2015).

This study had other findings similar to what is in the literature. We found that BPD patients had significantly higher comorbid unipolar major depression, consistent with the studies which reported approximately 75% of BPD patients have unipolar major depressive disorder (Gunderson et al., 2014; Perugi et al., 2013; Yoshimatsu & Palmer, 2014). We found that using the recommended cutoff score of ≥7 yielded the highest AUC for MDQ and MSI that were in line with the literature. The sensitivity (68% versus 73%) and specificity (84% versus 90%) of MDQ in our study were fairly similar to the original MDQ validation study (Hirschfeld et al., 2000). The specificity of MSI score in our study was somewhat higher than the pooled data from a recently published meta‐analysis (73% versus 66%)(Zimmerman, 2021), whereas the sensitivity was lower (63% versus 80%). This could be due to different study populations, inpatient versus combination (in the meta‐analysis) (Zimmerman, 2021).

The amalgamation of MSI+MDQ to diagnose BD or BPD did not appear to have a robust increase in accuracy based on ROC‐AUC, probably due to the overlap of symptomatology among both the disorders. Thus, focusing on a model which is based on the sublimes that were significant in differentiating both disorders showed promising results, most notably for BD. Although this model needs to be replicated in an independent sample with a Structured Clinical Interview (SCID)‐confirmed BD and BPD diagnoses, it has a merit of becoming a tool that can be used when the specific diagnosis of BD or BPD is unclear.

The strengths of this study include relatively large sample size, comparison of screening results with clinical diagnosis, and enrolling a consecutive sample of admitted adults to a specialized Mood Disorder Unit. Nonetheless, this study is limited by reliance on clinical diagnosis. Clinical diagnoses can be problematic in their reliability. All the diagnoses in this study were confirmed by Board‐Certified Psychiatrists based on extensive chart review and reviewing patient's diagnosis at discharge. In the absence of biological biomarkers, DSM diagnoses based on structured clinical interviews or research diagnoses based upon SCID are commonly used as reference standards. The prevalence of psychosis (3%) and the rates of comorbid substance use disorder (15%) were low in our study. The results might vary in an inpatient unit with different psychopathology (e.g., in patients with higher rates of substance use disorder and psychosis). Similarly, MDQ and MSI, as self‐report instruments, rely on the patients’ own retrospective recall of their symptoms, which could be influenced by the patients’ current memory, cognition, and mood, particularly in the setting of acute hospitalization.

## 5 CONCLUSION

These results offer practical and useful clinical guidance for focusing on specific subitems from the MDQ and MSI that can help distinguish BPD and BD. Misdiagnosis of these complex conditions leads to inappropriate management of the patients. Future studies utilizing SCID to replicate our findings in independent cohorts are encouraged. Further work with larger datasets and more rigorous bioinformatics machine learning methodology is encouraged to continue to identify distinguishing features of these two disorders to guide diagnostic precision and subsequent treatment recommendations.

## CONFLICT OF INTEREST

All authors declare that the research was conducted in the absence of any commercial or financial relationships that could be construed as a potential conflict of interest.

## Supporting information

Supporting information
