# Supplementary material for: Self‐report screening instruments differentiate bipolar disorder and borderline personality disorder
Source: Brain Behav. 2021 May 30;11(7):e02201. doi: 10.1002/brb3.2201 (PMC8323027; doi:10.1002/brb3.2201)

**Supplement.**

**Table S1.** Demographics characteristics /comorbidities of the cohort

| **Patient characteristics** | **N=757** |
| --- | --- |
| Age, mean (SD) | 40.8 (13.2) |
| Female, n (%) | 510 (67.4) |
| Psychiatric Comorbidities |  |
| Adjustment disorder | 38 (5.0%) |
| Anxiety disorders | 292 (38.6%) |
| Eating disorders | 26 (3.4%) |
| Impulse Control disorder | 19 (2.5%) |
| Psychotic disorders | 21 (2.8%) |
| Sleep disorders | 91 (12.0%) |
| Somatoform disorder | 32 (4.2%) |
| Substance Related disorders | 114 (15.1%) |
| Number of admissions | 1.14 (0.45%) |
| MDQ score, mean (SD) | 5.26 (3.8%) |
| MSI score, mean (SD) | 4.86 (2.6%) |
| MDQ+ve | 190 (25.0%) |
| MSI+ve | 225 (29.7%) |
| Borderline personality disorder | 60 (7.9%) |
| Bipolar disorder | 130 (17.2%) |
| Major depressive disorder | 532 (70.3%) |
| MDQ=Mood Disorder Questionnaire; MSI-BPD=McLean Screening Instrument for Borderline Personality Disorder; SD=standard deviation | |

**Table S2:** Cut-off point statistics for various MDQ scores predicting bipolar disorder, and MSI scores predicting borderline personality disorder.

|  | Cut-off points | Sensitivity | Specificity | AUC |
| --- | --- | --- | --- | --- |
| MSI | 8 | 0.654 | 0.872 | 0.758 |
|  | **7** | **0.678** | **0.837** | **0.763** |
|  | 4 | 0.754 | 0.699 | 0.726 |
|  | 6 | 0.700 | 0.804 | 0.752 |
|  | 3 | 0.754 | 0.640 | 0.697 |
|  | 5 | 0.715 | 0.777 | 0.746 |
|  | 2 | 0.754 | 0.604 | 0.679 |
| MDQ | 6 | 0.733 | 0.601 | 0.667 |
|  | 8 | 0.450 | 0.838 | 0.644 |
|  | **7** | **0.633** | **0.732** | **0.683** |
|  | 2 | 0.917 | 0.237 | 0.577 |
|  | 4 | 0.850 | 0.660 | 0.595 |
|  | 5 | 0.800 | 0.459 | 0.630 |
|  | 3 | 0.917 | 0.310 | 0.577 |

.

TableS-3. Individual level data for MDQ and MSI-BPD scales.

| MDQ individual level data | N (%) Present |
| --- | --- |
| Q1 | 109 (63.7%) |
| Q2 | 122 (70.9%) |
| Q3 | 109 (63.4%) |
| Q4 | 116 (67.8%) |
| Q5 | 126 (73.7%) |
| Q6 | 152 (88.9%) |
| Q7 | 144 (85.2%) |
| Q8 | 124 (72.5%) |
| Q9 | 119 (69.6%) |
| Q10 | 73 (42.4%) |
| Q11 | 88 (51.5%) |
| Q12 | 111 (64.5%) |
| Q13 | 87 (50.6%) |
| MSI individual level data | N (%) Present |
| Q1 | 105 (61.4%) |
| Q2 | 101 (59.1%) |
| Q3 | 121 (70.8%) |
| Q4 | 142 (83.0%) |
| Q5 | 109 (64.1%) |
| Q6 | 112 (66.3%) |
| Q7 | 64 (37.7%) |
| Q8 | 132 (79.0%) |
| Q9 | 78 (45.9%) |
| Q10 | 79 (46.2%) |
| Percentages are calculated based on the individual level data after excluding any missing data. | |

**Figure-S1.** Model performance ROC curves of significant individual MDQ (1, 3, 4, 5, 8, and 9) and MSI (2) items predicting Bipolar Disorder and Borderline Personality Disorder.


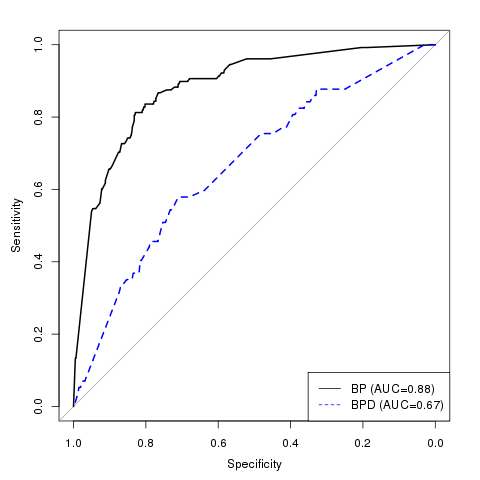

Supplement: Supplementary file 1 — Supporting information [file BRB3-11-e02201-s001.docx]
